# Performance Comparison of Laser and Ultrasonic Sensors for Water-Level Monitoring in Wind Turbine Towers: System Development and Experimental Validation

**DOI:** 10.3390/s26195996

**Published:** 2026-09-22

**Authors:** Jianchao Ma, Mingchen Wu, Jiarui Li, Xingzhu Ji, Nianyong Zhou

**Affiliations:** School of Urban Construction, Changzhou University, Changzhou 213164, China; majc@cczu.edu.cn (J.M.); wumingchenwkjjy@gmail.com (M.W.); lijiarui011@163.com (J.L.); 18385561913@163.com (X.J.)

**Keywords:** wind turbine tower, water depth monitoring, automatic drainage, sensor comparison, ultrasonic sensor, laser sensor, IoT

## Abstract

Water accumulation inside wind turbine towers threatens structural integrity and operational safety. This study developed an IoT-based water depth monitoring and automatic drainage system and evaluated the performance of laser (VL53L0X) and ultrasonic (HC-SR04) sensors for tower sump water depth measurement. Laboratory experiments investigated the effects of sensor-to-wall distance (10–150 mm) and water depth (10–350 mm) on measurement accuracy. The ultrasonic sensor is susceptible to side-wall interference within 100 mm due to its broad beam angle, while the laser sensor exhibits significant underestimation in shallow water below 100 mm due to optical penetration. In dynamic drainage tests, the ultrasonic sensor achieved complete water removal (<1 mm residual), whereas the standalone laser sensor left 55 mm of residual water. This limitation was mitigated by an opaque floating reflector, which enabled the laser sensor to achieve complete drainage. The developed system offers a cost-effective solution for unmanned tower sump monitoring, contributing to enhanced safety of wind turbine structures.

## 1. Introduction

Wind power has emerged as one of the fastest-growing renewable energy sources worldwide, with global installed capacity exceeding 1000 GW by the end of 2025 [1]; China alone accounts for over 40% of the global cumulative total [2]. However, the safe and stable operation of wind turbines continues to face persistent threats, among which water accumulation inside the tower has been identified as a particularly critical issue [3,4,5]. Water ingress into wind turbine towers can occur through multiple pathways, including rainwater seepage at flange connections, door openings, and foundation ring gaps, as well as groundwater intrusion through cracks in the concrete foundation [6,7]. Once water accumulates inside the tower, it accelerates corrosion of the steel structure and bolted connections, ultimately compromising the long-term structural integrity of the tower [8,9,10]. Once accumulated, water accelerates corrosion of the steel tower base and bolted connections, reduces the insulation resistance of power cables and electrical components, and significantly increases operation and maintenance costs due to unplanned downtime and structural repairs [11,12]. To mitigate water ingress, various waterproofing measures have been implemented in practice, such as applying sealants at flange joints, installing rubber gaskets around door frames, and coating the foundation ring with anti-corrosion materials [3,13]. However, wind turbines are typically installed in harsh outdoor environments and are subject to continuous low-frequency vibrations during operation, which gradually degrade sealing performance and render passive waterproofing insufficient over the long term [14,15]. Consequently, developing an active water-depth-monitoring and automatic drainage system has become essential for reducing residual risks.

A variety of water-depth-monitoring techniques have been explored and can be broadly categorized into contact-based and non-contact-based methods [16,17,18]. Contact-based methods rely on physical sensors such as float switches, pressure transducers, and electrode-type probes that directly contact the water surface [18]. Although simple and cost-effective, these methods suffer from fouling by suspended debris, vulnerability to corrosion, mechanical wear, and high maintenance requirements, making them unsuitable for long-term unattended monitoring in harsh field environments [15,19,20]. Non-contact methods, by contrast, have attracted growing attention due to their ability to operate without direct water contact, thereby avoiding corrosion and fouling. This trend aligns with the broader movement toward automated and data-driven Structural Health Monitoring (SHM), in which robotic platforms and embedded sensing systems are increasingly deployed for continuous infrastructure assessment [21,22]. Recent reviews have highlighted the potential of unmanned aerial vehicles (UAVs), wall-climbing robots, and AI-driven inspection systems for damage detection and real-time diagnostics while also identifying practical barriers related to system readiness, environmental constraints, and regulatory support [21]. A recent survey of construction professionals further identified accessibility and safety, regulatory support, and data-driven decision-making as the most influential factors determining the adoption of automated monitoring technologies [22]. Similar considerations apply to the deployment of low-cost non-contact sensors in confined industrial environments such as wind turbine towers, where installation constraints, maintenance access, and long-term reliability must be carefully balanced. Among non-contact methods, ultrasonic and laser sensors operating on the time-of-flight (ToF) principle have been widely adopted for water depth monitoring [23,24]. Although machine vision-based approaches have also been explored, their robustness under varying lighting conditions and high computational cost have hindered practical deployment in embedded systems [25]. Therefore, ultrasonic and laser sensors remain the preferred choices for autonomous water depth monitoring.

The measurement accuracy of ultrasonic and laser sensors has been extensively studied, with factors such as ambient temperature, installation height, and water turbidity identified as significant contributors to measurement errors [26,27,28,29,30]. For instance, Ranieri et al. [28] reported that increasing water turbidity reduces the measurement error of laser sensors by attenuating optical penetration through the water surface. Gul et al. [30] found that both installation height and ambient temperature positively correlate with measurement error and proposed a dual-sensor correction method incorporating rainfall and ultrasonic measurements to compensate for environmental effects. However, most existing studies have been conducted in open-water environments such as rivers and reservoirs, where sensors have unobstructed line-of-sight to the water surface. In contrast, the wind turbine tower sump presents a confined space with vertical walls in close proximity, potentially causing side-wall reflections that interfere with measurements. Given that ultrasonic sensors typically have beam angles of 15–30°, significantly broader than laser sensors (<1°), they may exhibit markedly different sensitivities to wall proximity [18,31]. Furthermore, due to the distinct physical properties of sound and light, ultrasonic waves are almost entirely reflected at the air–water interface owing to extreme acoustic impedance mismatch, while infrared laser beams can partially penetrate clear shallow water and reflect off the bottom, leading to water depth underestimation [28,32]. Despite these critical differences, limited research has systematically compared the performance of ultrasonic and laser sensors specifically for water depth monitoring inside wind turbine towers.

In related fields, non-contact sensors have been employed for water depth monitoring in drainage pipes and sewer systems [16,23,27,33,34]. For example, Zhu et al. [16] developed low-cost Internet of Things (IoT)-based blockage detection systems using ultrasonic sensors and evaluated their accuracy under various flow conditions. However, none of these studies specifically addressed the influence of vertical walls on sensor accuracy in confined tower-like geometries, nor did they investigate dynamic drainage processes where water depth continuously decreases to near-zero depths. The extent to which wall distance and water depth affect sensor accuracy in tower sump applications remains largely unexplored.

To address this research gap, the present study develops an IoT-based water-depth-monitoring and automatic drainage system specifically designed for wind turbine towers. The performance of laser (VL53L0X) and ultrasonic (HC-SR04) sensors is systematically evaluated through laboratory experiments focusing on two key factors: sensor-to-wall distance and water depth. Dynamic drainage tests are conducted to assess the real-time tracking capability of each sensor during pump operation, and an opaque floating reflector is proposed and validated as a cost-effective solution to overcome the shallow-water limitation of the laser sensor. The novelty of this work lies in two aspects. First, it provides a systematic comparison of laser and ultrasonic sensors in a confined tower-like geometry, where wall proximity and shallow water conditions differ fundamentally from open-water environments. Second, it proposes and validates a simple, low-cost solution (an opaque floating reflector) that enables the laser sensor to achieve complete drainage in shallow water. The findings provide practical guidelines for sensor selection and installation in tower sump monitoring systems, contributing to enhanced corrosion prevention and operational safety of wind power structures.

## 2. Materials and Methods

### 2.1. System Design and Hardware Configuration

To systematically evaluate the performance of different sensors for water depth monitoring and automatic drainage in wind turbine towers, a dedicated monitoring and control system was developed. The system architecture and main hardware components are illustrated in Figure 1, which comprises four functional units: (a) sensing unit, (b) control and communication unit, (c) power supply unit, and (d) execution unit.

Two types of commercially available ranging sensors were employed for comparative evaluation: a laser sensor (VL53L0X, STMicroelectronics, Geneva, Switzerland) with an operating wavelength of 940 nm, and an ultrasonic sensor (HC-SR04, generic) with an operating frequency of 40 kHz. Both sensors were interfaced with an ESP32 microcontroller (S3N16R8, Espressif Systems, Shanghai, China) for real-time data acquisition and processing. A mini self-priming water pump (385-C, 12 V DC, generic) was used for water drainage, activated by a relay module controlled by the ESP32 based on the measured water depth. A power monitoring module (INA219, Texas Instruments, Dallas, TX, USA) was integrated to continuously record the pump’s operating current and voltage for diagnostic purposes. The entire system was powered by a 12 V DC supply, with a step-down voltage regulator (LM2596S, generic) providing 5 V for the ESP32 and its peripheral components. Data acquired by the controller can be transmitted wirelessly via Wi-Fi or Bluetooth to a remote computer or mobile device, enabling real-time monitoring and control. The detailed system schematic and hardware configuration are presented in Figure 1a and Figure 1b, respectively.

The proposed system is intended for both new wind turbine installations and retrofit applications on existing towers. In new installations, the sensor bracket, controller, and pump can be integrated into the tower sump during construction. In retrofit applications, the system can be installed without modifying the tower structure, making it suitable for towers that have exhibited water accumulation problems or are located in regions with high rainfall or high groundwater levels.

For practical deployment, the sensors, the control unit, and the pump are mounted on a rigid platform fixed to the inner wall of the tower sump. The sensors are installed with the head facing downward toward the water surface, and their installation height can be set within the sensor’s specified operating range to accommodate different tower sump geometries. In practical deployment, the height should be adjusted according to the tower sump depth and the sensor’s measurement range, while ensuring sufficient wall clearance for the ultrasonic sensor to avoid side-wall interference. The minimum required clearance is determined by the beam geometry and is experimentally evaluated in Section 3.1. The signal and power cables are routed along the tower wall to the control unit, which contains the ESP32, relay module, INA219 power monitor, and power supply. The pump is mounted on the same platform, with its intake connected to a suction pipe extending downward to the lowest point of the sump, and its discharge hose routed to a drainage outlet at the tower base. When a laser sensor is used, an opaque floating reflector is deployed on the water surface to provide a stable reflective interface. Figure 1c presents a conceptual schematic of the on-site installation scheme.

The communication performance of the system was evaluated by conducting an Internet Control Message Protocol (ICMP) ping test between a laptop and the ESP32 over the Wi-Fi network in the laboratory. A total of 1000 packets were sent, and the packet loss rate, round-trip latency (minimum, maximum, and average), reconnection time after intentional signal interruption, and Received Signal Strength Indicator (RSSI) were recorded.

### 2.2. Experimental Setup

All experiments were conducted in the Indoor Environmental Laboratory at Changzhou University, with ambient temperature maintained at 25 ± 2 °C and relative humidity at 50 ± 5%. A cylindrical opaque plastic container with an inner diameter of 300 mm and a height of 400 mm was used to simulate the tower interior. Both sensors were fixed on an adjustable bracket that allowed for precise control of the horizontal position (distance from the wall) and vertical position (installation height) of each sensor. The installation height of both sensors was fixed at 400 mm above the container bottom for all experiments, as shown in Figure 2.

To examine the wall proximity effect, the sensors were mounted at varying distances from the vertical container wall, ranging from 10 mm to 150 mm at 10 mm intervals. During these tests, the container was empty (i.e., no water was present), and the sensors measured the straight-line distance to the container bottom, which was fixed at 400 mm from the sensor. This configuration ensured that any deviation in the measured distance could be attributed solely to side-wall interference, independent of water depth effects.

To investigate the effect of water depth on sensor accuracy, both sensors were positioned at the center of the container (150 mm from the wall) to eliminate wall interference. The actual water depth was varied from 10 mm to 350 mm, and each water depth was maintained for at least 30 s prior to data recording to ensure stabilization.

To evaluate the system performance under laboratory-simulated tower sump condition, dynamic drainage tests were conducted starting from an initial water depth of 150 mm. The pump was activated and left running continuously until the sensor-reported water depth reached zero, at which point the pump was deactivated. The water depth was continuously recorded by both sensors simultaneously at 0.25 s intervals until the container was completely drained. Each drainage cycle lasted approximately 360 s. During the drainage process, the actual water level was manually recorded at 10 s intervals using a calibrated steel ruler with a resolution of 1 mm. These manually recorded values were used as the reference for all error calculations. Two configurations were tested: (1) the standalone laser sensor as the feedback element for pump control, and (2) the laser sensor with an opaque floating reflector deployed on the water surface. The ultrasonic sensor was monitored simultaneously in both configurations for comparison, but was not used as the reference for error calculation. The residual water depth after pump deactivation was measured and recorded for each configuration to quantify the drainage completeness.

The opaque floating reflector was fabricated from an expanded polystyrene (EPS) disc with a diameter of 300 mm (matching the inner diameter of the simulated tower container), a thickness of 5 mm, and a density of approximately 25 kg/m^3^. Its buoyancy was sufficient to keep the reflector floating on the water surface with the top surface approximately 2 mm above the waterline, ensuring continuous contact with the water surface even at very shallow depths. The EPS material is opaque to the infrared laser beam at 940 nm, thereby providing a distinct and stable reflective interface for the laser beam and preventing optical penetration into the water.

The system supports user-configurable activation and deactivation thresholds for the pump. In this study, the activation threshold was set to 150 mm and the deactivation threshold to 0 mm. Once activated, the pump runs continuously until the sensor-reported water depth reaches zero, indicating that the sump has been emptied. No intermediate thresholds or hysteresis bands were applied, because the pump operates continuously from activation to complete drainage without intermediate cycling. The water depth is sampled at 0.25 s intervals, and the relay switching time is approximately 10 ms. The control logic is implemented on the ESP32.

### 2.3. Measurement Principles and Error Metrics

The distance from the sensor to the water surface is determined by the time-of-flight principle [35]:

(1) $$D_{sensor}=\frac{v\times ∆t}{2}$$

where *D*_sensor_ is the distance from the sensor to the target surface measured by the sensor (mm), *v* is the speed of sound in air (approximately 343 m/s at 25 °C) for the ultrasonic sensor, or the speed of light for the laser sensor, and Δ*t* is the round-trip flight time (s). In the wall proximity tests, the target surface is the container bottom; in the water depth tests, the target surface is the water surface.

Two types of errors were defined corresponding to the two experimental scenarios. For the wall proximity tests, the measurand is the distance from the sensor to the container bottom, and the ranging error *E*_D_ and relative error *RE*_D_ are defined as follows:

(2) $$E_{D}=D_{sensor}-D_{actual}$$

(3) $$RE_{D}=\frac{E_{D}}{D_{actual}}\times 100\%$$

where *D*_actual_ = 400 mm is the true sensor-to-bottom distance. A positive *E*_D_ indicates that the measured distance is longer than the true distance.

For the water depth tests, the measurand is the water depth, which is derived from the measured distance via:

(4) $$H=H_{instal}-D$$

where *H* is the water depth above the container bottom (mm), *H*_inst_ is the sensor installation height relative to the container bottom (mm), fixed at 400 mm in this study, and *D* is the distance from the sensor to the target surface measured by the sensor (mm).

The water depth error *E*_H_ and relative error *RE*_H_ are defined as follows:

(5) $$E_{H}=H_{sensor}-H_{actual}$$

(6) $$RE_{H}=\frac{E_{H}}{M_{actual}}\times 100\%$$

where *H*_sensor_ is the water depth reported by the sensor (mm), *H*_actual_ is the true water depth measured by a calibrated steel ruler (mm). A negative *E_H_* indicates that the reported water depth is lower than the actual depth.

Since *H*_inst_ is constant, the two error types are related by *E_H_* = −*E_D_*. It should be noted that the denominators differ: *RE*_D_ uses the fixed installation height (400 mm), whereas *RE*_H_ uses the actual water depth, which varies from 10 to 350 mm.

Unless otherwise emphasized, *E_H_* and *E_D_* refer to mean errors over three independent replicates, and *RE*_D_ and *RE*_H_ refer to mean relative errors. Any systematic offset identified during calibration was corrected in the firmware to ensure initial accuracy and consistency across all measurements.

For each test condition, the measurement lasted 360 s with a recording interval of 0.25 s, yielding 1440 data points per condition. For each run, the mean value of all recorded data points was calculated, and these three run-level means were treated as independent replicates (*n* = 3) for statistical analysis. The mean, standard deviation (SD), root mean square error (RMSE), and mean absolute error (MAE) were calculated as follows:

(7) $$Mean=\frac{1}{n}\sum_{i=1}^{n}\bar{x_{i}}$$

(8) $$SD=\sqrt{\frac{1}{n-1}\sum_{i=1}^{n}(\bar{x_{i}}-Mean{)}^{2}}$$

(9) $$RMSE=\sqrt{\frac{1}{n}\sum_{i=1}^{n}(\bar{x_{i}}-x_{ture}{)}^{2}}$$

(10) $$MAE=\frac{1}{n}\sum_{i=1}^{n}\left|\bar{x_{i}}-x_{ture}\right|$$

where x_i_ is the mean value of the i-th run, *n* = 3, and x_ture_ is the true value. The SD across the three replicate means also represents the repeatability of the measurement.

## 3. Results and Discussion

### 3.1. Effect of Tower Wall Proximity on Sensor Accuracy

To evaluate the influence of the tower wall on sensor measurements, the distance between the sensor and the vertical wall was varied from 10 mm to 150 mm at 10 mm intervals. Figure 3 presents the measured distances and corresponding errors for both sensors under different wall proximity conditions. Figure 3a–c show the raw distance readings of both sensors at wall distances of 10 mm, 50 mm, and 100 mm, respectively. The ultrasonic sensor exhibited pronounced measurement fluctuations when positioned at 10 mm and 50 mm from the wall, with measured distances to the water surface exceeding the actual sensor installation height of 400 mm. This indicates that the ultrasonic signal was subject to significant side-wall interference under these conditions. In contrast, the laser sensor maintained stable readings with minimal fluctuations across all wall distances, demonstrating its insensitivity to wall proximity.

Figure 3d and Figure 3e present the mean ranging error *E_D_* and mean relative ranging error *RE_D_* as functions of wall distance, respectively. For the ultrasonic sensor, *E_D_* decreased monotonically as the wall distance increased, approaching zero at a threshold of approximately 100 mm. At a wall distance of 10 mm, the ultrasonic sensor yielded a mean ranging error of *E_D_* = +11 mm (corresponding to *RE_D_* = 3%), while the maximum instantaneous error reached +45.6 mm (corresponding to *RE_D_* = 11%). The large gap between the mean and maximum values reflects the pronounced fluctuations caused by multi-path interference near the wall. Beyond 100 mm, wall interference became negligible. In contrast, the laser maintained *E_D_* = 0 across all wall distances, demonstrating no observable correlation with wall proximity.

These results indicate that, under the tested configuration (HC-SR04, installation height 400 mm, container diameter 300 mm), a minimum clearance of approximately 100 mm from the wall was required for the ranging error *E*_D_ to become 0. This threshold is defined by the criterion that the sensor reading equals the true distance, rather than by an arbitrary error limit. It is governed by the beam footprint on the target surface, which depends on the installation height *H*_inst_ and and the effective beam angle *θ*. The single-side beam radius is *L*_half_ = *H*_inst_ × tan (*θ*/2). For the HC-SR04 at 400 mm installation height, this yields approximately 53 mm for *θ* = 15° and 107 mm for *θ* = 30°. The observed threshold of approximately 100 mm is consistent with the wider effective beam angle of 30°. If the installation height were doubled to 800 mm, the threshold would scale linearly to approximately 200 mm. If a sensor with a narrower beam angle were used, the threshold would decrease accordingly. If the container wall were made of steel instead of plastic, stronger acoustic reflections could require a larger clearance. Therefore, this 100 mm value is specific to the tested sensor, installation height, and container material and should not be generalized without re-evaluation.

Conversely, the laser sensor features a much narrower beam divergence (typically <1°), which effectively confines the emitted infrared beam to a concentrated path [18,31]. The narrow beam ensures that the optical signal interacts almost exclusively with the water surface directly beneath the sensor, rendering it virtually insensitive to side-wall reflections. This inherent immunity to nearby obstacles makes laser sensors a robust choice for installations where spatial constraints preclude maintaining a 100 mm clearance from the tower wall.

The statistical analysis of the ranging error across three independent replicates is summarized in Table 1. At a wall distance of 10 mm, the ultrasonic sensor yielded a mean error of *E*_D_ = 11 mm with a standard deviation (SD) of 4 mm, and the SD decreased to within 1 mm as the wall distance increased to 100 mm and beyond. The laser sensor maintained a mean error of 0 mm and an SD of 0 mm across all wall distances, confirming its superior repeatability and insensitivity to wall proximity.

### 3.2. Effect of Water Depth on Sensor Accuracy

To evaluate the influence of water depth on sensor accuracy, both sensors were tested across a water depth range of 10 mm to 350 mm. Figure 4 presents the mean water depth error *E*_H_ and mean relative error *RE*_H_ as functions of the actual water depth.

When the water depth exceeded 100 mm, both sensors performed satisfactorily, with error values *E* close to 0 mm. However, a pronounced performance divergence emerged when the water depth fell below 100 mm. The ultrasonic sensor maintained excellent accuracy throughout the shallow water range, while the laser sensor exhibited substantial negative errors, with *E_H_* = −8 mm and *RE_H_* = −80% at the 10 mm depth. The laser sensor’s deviations became increasingly severe as water depth decreased, with the largest errors concentrated in the 0–100 mm range. These results indicate that the ultrasonic sensor is reliable for shallow water monitoring, whereas the laser sensor requires auxiliary measures to ensure accurate detection under near-empty conditions.

For the laser sensor, the substantial negative errors (i.e., water depth underestimation) in shallow water originate from optical penetration through the water surface. When the infrared beam impinges on a clear and shallow water layer, a significant portion of photons transmits through the air–water interface rather than reflecting at the surface [24]. These transmitted photons travel through the water column and reflect off the container bottom, resulting in an extended round-trip path. The sensor interprets this longer flight time as a greater distance to the reflective surface, which yields a reported water depth lower than the actual value. This penetration effect becomes pronounced when the water depth falls below approximately 100 mm, which is the depth at which the water depth error *E*_H_ becomes zero in the tested configuration. Above this depth, the sensor reading equals the true water depth. Below this depth, the sensor exhibits significant negative errors due to optical penetration. This threshold depends on the ratio of light reflected at the water surface to that transmitted through the water and reflected from the bottom, which is governed by the Fresnel reflectance at the air–water interface, the water clarity, and the bottom reflectivity. It does not depend significantly on installation height. If the water were turbid, the penetration would be attenuated and the threshold would shift to a smaller water depth. If the bottom were more reflective, the error would be amplified and the threshold would shift to a larger water depth. These trends are based on the physical mechanism but have not been experimentally validated in this study.

The physical mechanism of the laser error was analyzed quantitatively. For light at normal incidence, the Fresnel reflectance at the air–water interface is given by *R* = ((*n*_1_ − *n*_2_)/(*n*_1_ + *n*_2_))^2^ where *n*_1_ = 1 for air and *n*_2_ = 1.33 for water at 940 nm [36]. This yields *R* = 0.02, indicating that only about 2% of the incident light is reflected at the water surface, while approximately 98% is transmitted into the water. The transmitted light reflects off the container bottom and returns to the sensor, resulting in an apparent distance of *D*_sensor_ = (*H*_instal_ − *H*_actual_) + *n*·*H*_actual_, where *H*_instal_ − *H*_actual_ is the true air gap between the sensor and the water surface, *n* = 1.33 is the refractive index of water, and *H*_actual_ is the actual water depth. The factor *n*·*H*_actual_ accounts for the approximately 25% reduction in the speed of light in water compared with air. Since *D*_sensor_ > *H*_instal_ − *H*_actual_, the sensor reports a water depth *H*_sensor_ = *H*_instal_ − *D*_sensor_ that is lower than *H*_actual_, yielding a negative water depth error *E*_h_ < 0. For the tested condition of *H*_actual_ = 10 mm, the predicted water depth error is *E*_H_ = −3.3 mm, which is of the same sign and order of magnitude as the measured error of *E*_H_ = −8 mm. The remaining discrepancy is attributed to additional optical effects such as partial reflection from suspended particles and multiple reflections within the water layer. Similar shallow-water underestimation artifacts caused by optical penetration and reduced light speed in water have been reported in laser bathymetry and airborne LiDAR studies [24,37].

For the ultrasonic sensor, the excellent accuracy in shallow water is attributed to the extreme acoustic impedance mismatch at the air–water interface. An impedance ratio of approximately 1:3500 between air and water renders the measurement virtually insensitive to water clarity or water depth [38,39]. This inherent property makes ultrasonic sensing reliable for shallow water detection.

The practical implication is critical for tower sump drainage. The laser sensor’s water depth underestimation in the 0–100 mm zone would cause premature pump deactivation, leaving residual water that promotes long-term corrosion. To overcome this limitation, an opaque floating reflector was deployed on the water surface to provide a distinct reflective interface for the laser beam. The performance of this enhanced configuration is evaluated in Section 3.3.

The statistical analysis of the water depth error across three independent replicates is summarized in Table 2. The ultrasonic sensor maintained a mean error within ±1 mm and an SD below 1 mm across the entire tested depth range, confirming its excellent accuracy and repeatability in shallow water. In contrast, the laser sensor exhibited a mean error of −8 mm with an SD of 2 mm at a water depth of 10 mm, and the error decreased progressively to near zero as the water depth increased to 150 mm and beyond.

### 3.3. Drainage Performance Comparison

To evaluate the practical drainage performance of the monitoring system, the pump was activated to drain water from the simulated tower sump starting from an initial water depth of 150 mm. The actual water depth was simultaneously measured using the steel ruler for reference (resolution 0.5 mm). Figure 5a presents the drainage curves of both the ultrasonic-based and standalone laser-based systems, and Figure 5b shows the drainage performance of the laser sensor with the floating reflector in comparison with the ultrasonic sensor.

As shown in Figure 5a, when the ultrasonic sensor served as the feedback element, the reported water depth closely tracked the manually recorded reference values throughout the entire drainage process. The pump was reliably deactivated when the water depth approached zero, achieving complete water removal with a residual depth of less than 1 mm. By contrast, the standalone laser sensor exhibited a significant deviation during the shallow-water phase, reporting water depth lower than the actual values, which corresponds to an underestimation of the water depth. This caused the control system to prematurely deactivate the pump, leaving a residual water layer of approximately 55 mm in the container after the drainage cycle.

When the opaque floating reflector was deployed on the water surface to provide a distinct reflective interface for the laser beam, the drainage performance improved markedly, as shown in Figure 5b. With this enhancement, the laser sensor successfully tracked the water depth throughout the entire drainage process, and the reported water depth closely matched the actual level across all depths. The pump was deactivated when the water depth reached zero, achieving complete water removal comparable to that of the ultrasonic-based system (<1 mm residual depth).

The standalone laser sensor’s failure to achieve complete drainage stems from its optical penetration artifact in shallow water. As the water depth decreased from 150 mm to approximately 55 mm, the sensor progressively underestimated the water depth, creating a “false empty” condition while a significant water layer remained. In wind turbine towers, even a thin residual water film can initiate or accelerate corrosion processes at the steel-concrete interface of the tower base; a residual layer of 55 mm represents a substantial and unacceptable risk.

The floating reflector effectively addresses the optical penetration issue by shifting the reflective interface from the partially transparent water surface to an opaque solid surface. The reflector’s buoyancy ensures continuous contact with the water surface regardless of the water depth, providing a reliable target for the laser sensor even in extremely shallow conditions. With this enhancement, the laser sensor achieves complete drainage comparable to the ultrasonic sensor, making it a viable alternative for installations where wall proximity constraints preclude ultrasonic deployment or where higher measurement resolution is desired.

It is worth noting that the floating reflector introduces additional mechanical considerations for practical deployment, including appropriate sizing to prevent jamming against the tower walls, material selection for chemical resistance to potentially corrosive sump environments, and long-term buoyancy stability. Despite these engineering considerations, the floating reflector offers a simple, low-cost, and effective solution that enables the use of laser sensors in tower sump drainage applications.

The different sensor responses during drainage can be understood from the physical nature of the measurements. The saw-tooth pattern in the ultrasonic curve is consistent with the combined effects of water surface fluctuations (induced by pump suction and outflow) and the internal averaging of multiple echo signals within the sensor. Previous studies have reported similar jitter and fluctuation in ultrasonic level measurements under turbulent conditions [23,40,41]. Because the ultrasonic beam has a relatively wide angle, the received echo may represent an average over a finite area of the fluctuating water surface, resulting in small periodic oscillations in the measured distance. In contrast, the standalone laser sensor exhibited occasional upward spikes in the reported water depth, corresponding to abrupt decreases in the measured distance. Under normal conditions, a large fraction of the infrared light is transmitted through the clear water and reflects off the container bottom, resulting in an overestimated distance and underestimated water depth. However, previous studies have shown that bubbles or surface disturbances can cause the laser to receive reflections from submerged bubbles or from the water surface itself, leading to abrupt changes in the measured distance [24]. These transient spikes are consistent with such momentary changes in the reflection condition. When the opaque floating reflector is deployed, the reflection interface is fixed at the reflector surface, which suppresses the interface switching and largely removes the associated spikes and abrupt changes.

Both sensor types can be effectively employed for water depth monitoring in wind turbine towers, provided that their respective installation requirements are carefully addressed. The ultrasonic sensor is recommended for direct shallow-water monitoring where spatial constraints permit a clearance of 100 mm in the tested configuration, while the laser sensor with an opaque floating reflector is suitable for installations where space is limited or higher measurement resolution is desired.

### 3.4. Communication Performance

The Wi-Fi communication test yielded a packet loss rate of 0% (1000 packets sent, 1000 received), with a minimum round-trip latency of 1 ms, a maximum of 40 ms, and an average of 4 ms. After intentional signal interruption, the ESP32 automatically reconnected within 4.83 s. The RSSI was −69 dBm, indicating a stable connection. These results demonstrate that the wireless communication link is reliable and that the average latency of 4 ms is well below the 250 ms sampling interval (4 Hz) used in this study. Therefore, the communication performance is adequate for real-time water depth monitoring under laboratory conditions.

## 4. Limitations and Future Work

The laboratory setup is a simplified representation of a real wind turbine tower sump. The 300 mm container used in this study is smaller than a full-scale tower base. However, the wall distances tested in this study (10–150 mm) were selected to cover the range where wall interference is most severe. In practice, the sensor is mounted on a bracket attached to the inner wall, and the distance from the wall depends on the bracket design and available mounting space. Therefore, the laboratory results represent a conservative assessment of wall interference. For installations where the sensor can be positioned farther from the wall, the interference effect is expected to be smaller or negligible. In contrast, the shallow-water optical penetration effect of the laser sensor depends on water depth rather than container diameter, and is therefore expected to persist in full-scale tower sumps. The floating reflector solution is likewise independent of tower dimensions and remains applicable.

Several factors specified as critical for field applications have not been validated in this study, including water turbidity, oil film and foam, temperature variation, tower vibration, and long-term corrosion. These factors may affect sensor performance through different mechanisms: turbidity may attenuate the laser beam and reduce optical penetration; oil films and foam may create false reflective interfaces for both sensors; temperature variation may alter the speed of sound and the laser electronics; tower vibration may affect sensor mounting stability and water surface conditions; and long-term corrosion may alter wall reflectivity and water chemistry. In addition, steel wall reflection, foundation geometry, inclination, and water surface disturbance were not replicated. These factors should be addressed in future field tests under actual tower conditions.

The two 100 mm thresholds identified in this study are specific to the tested sensor models (HC-SR04 and VL53L0X), the vertical downward mounting orientation, the opaque plastic container with a 300 mm inner diameter, the 400 mm installation height, and clean tap water at controlled temperature and humidity. They were defined using the criterion that the measurement error decreased to within ±1 mm. For different sensor models, beam angles, installation heights, container materials, water turbidity, or bottom reflectivity, these thresholds should be re-evaluated. In practical tower installations, the required ultrasonic wall clearance should be calculated from the beam geometry, and the laser shallow-water limitation should be addressed by deploying an opaque floating reflector.

The true water level in the dynamic drainage experiments was obtained by manual reading of a calibrated steel ruler (resolution 1 mm) at 10 s intervals. Although this provides discrete validation points, it cannot fully resolve the continuous dynamic curve sampled at 0.25 s. In addition, the ultrasonic sensor was used only as a comparison sensor and not as a reference. Future work should employ an independent continuous water-level sensor, such as a pressure transducer or a weighing-based system, to provide a fully continuous benchmark for dynamic drainage validation.

The communication performance was evaluated only under laboratory conditions over a short distance and without steel enclosure, mechanical vibration, or electromagnetic interference from turbine electrical systems. The communication reliability, latency, and long-term stability under actual tower operational conditions have not been validated and remain to be investigated in future work.

## 5. Conclusions

This study developed an IoT-based water-depth-monitoring and automatic drainage system for wind turbine towers, with a systematic comparative evaluation of laser and ultrasonic sensors under simulated tower sump conditions. The main contributions of this work are twofold. First, it provides the first systematic comparison of laser and ultrasonic sensors in a confined tower-like geometry, demonstrating that wall proximity and shallow water conditions produce error mechanisms that are absent in open-water environments. Second, it proposes and validates an opaque floating reflector that eliminates the laser sensor’s shallow-water limitation, enabling complete drainage comparable to the ultrasonic system. The following conclusions are drawn:

(1) The ultrasonic sensor is susceptible to side-wall interference when positioned close to the tower wall due to its broad beam angle, which causes multi-path reflections and overestimated ranging distances. In the tested configuration (HC-SR04, effective beam angle approximately 30°, vertical downward mounting, 400 mm installation height, 300 mm plastic container), the ranging error *E*_D_ became zero only when the wall clearance exceeded approximately 100 mm. This threshold is governed by the beam footprint and scales with installation height and beam angle; it should not be treated as a universal rule. In practical installations, the required clearance can be estimated from the single-side beam radius *L*_half_ = *H*_inst_ × tan (*θ*/2). For different sensor models, installation heights, or container materials, the threshold should be re-evaluated accordingly.
(2) The laser sensor exhibits significant water depth underestimation when the water depth falls below approximately 100 mm under the tested conditions (VL53L0X, vertical downward mounting, 400 mm installation height, 300 mm plastic container, clean tap water). This threshold corresponds to the depth at which the water depth error *E*_H_ becomes zero; above this depth, the sensor reading equals the true water depth. The underestimation originates from optical penetration through the air–water interface, where transmitted photons reflect off the container bottom rather than the water surface, artificially extending the measured flight path. This threshold depends on water clarity and bottom reflectivity. If the water were turbid, the penetration would be attenuated and the threshold would shift to a smaller water depth; if the bottom were more reflective, the error would be amplified and the threshold would shift to a larger water depth. These trends are based on the physical mechanism but have not been experimentally validated. The proposed opaque floating reflector effectively mitigates this limitation by providing a distinct reflective interface for the infrared beam, enabling complete water removal comparable to the ultrasonic-based system, and is recommended as a robust solution for practical installations.
(3) Under the tested laboratory conditions, both sensor configurations can be effectively employed for tower sump drainage when their installation requirements are met. The ultrasonic sensor is recommended for shallow-water monitoring where sufficient wall clearance is available, while the laser sensor with an opaque floating reflector is suitable for confined spaces. The floating reflector is particularly robust because it does not rely on the shallow-water threshold being satisfied.

These recommendations are specific to the tested sensor models, container geometry, and water conditions. They should not be generalized to other wind turbine tower applications without re-evaluation and field validation. Future work will include field testing under actual turbine tower operational conditions, long-term reliability assessment, and integration with existing wind turbine Supervisory Control and Data Acquisition (SCADA) systems for automated condition-based maintenance.

## Figures and Tables

**Figure 1:**
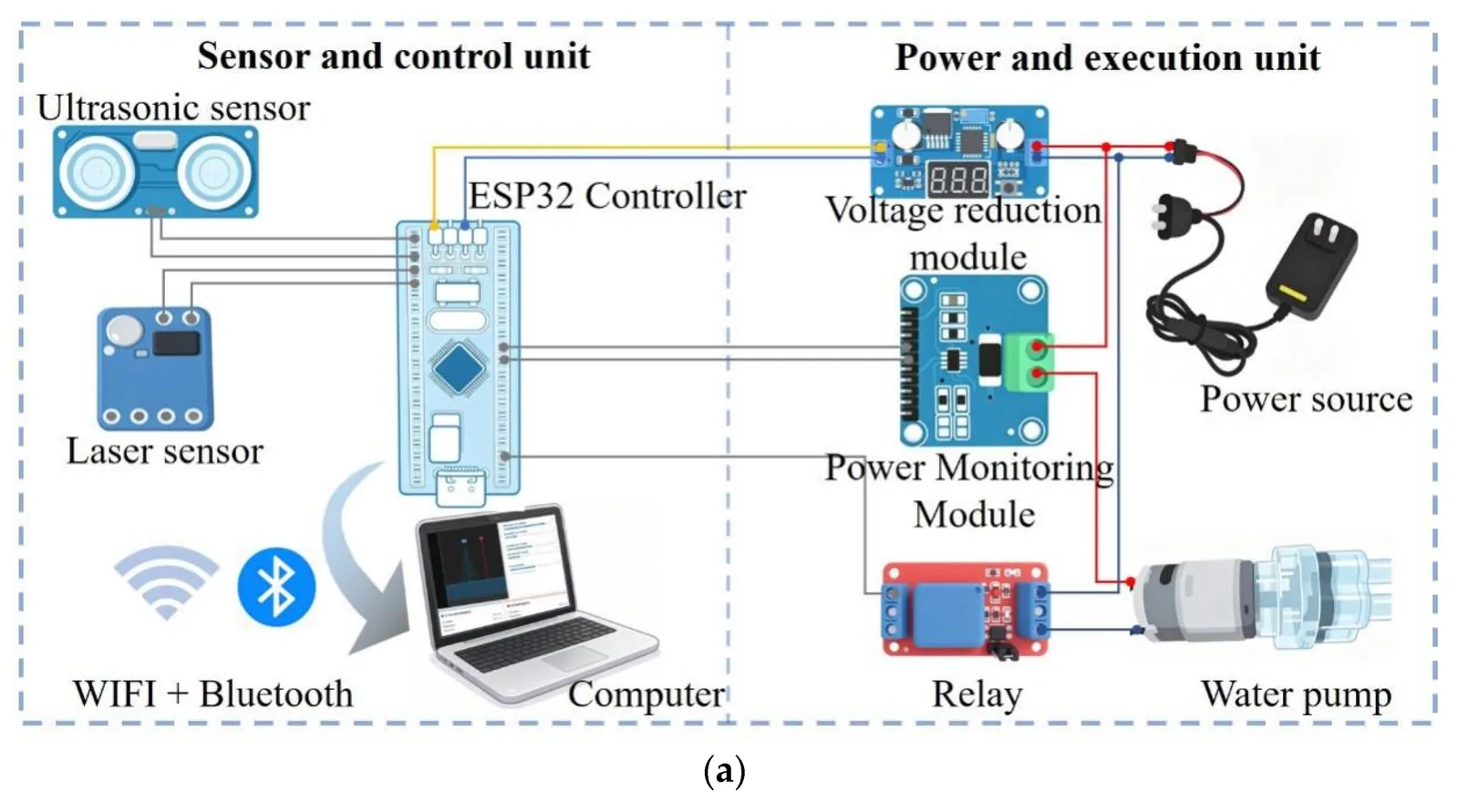

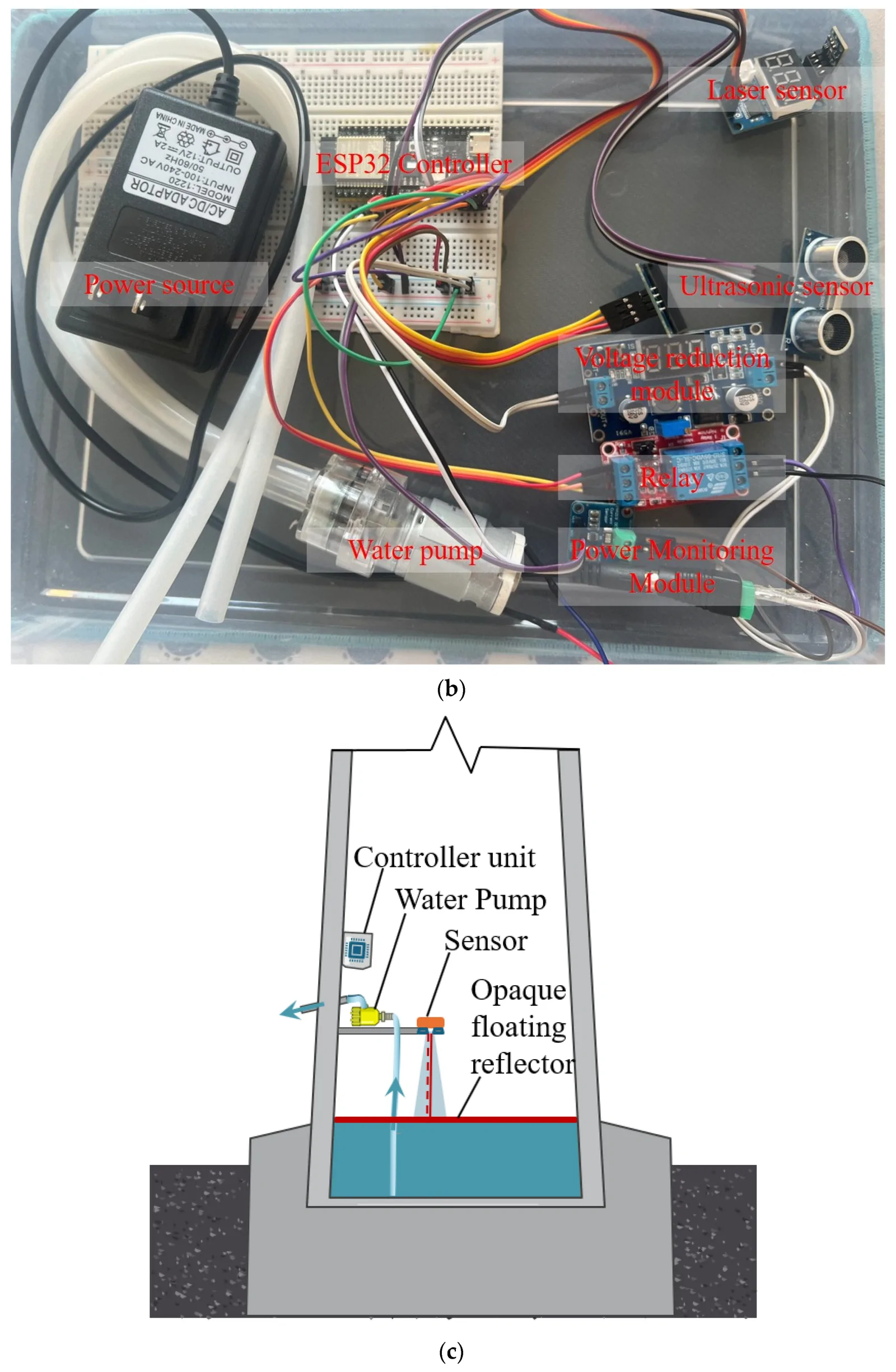
Schematic diagram and hardware implementation of the water-depth-monitoring and automatic drainage system for wind turbine towers: (**a**) Schematic diagram; (**b**) Hardware implementation; (**c**) Conceptual schematic of on-site installation.

**Figure 2:**
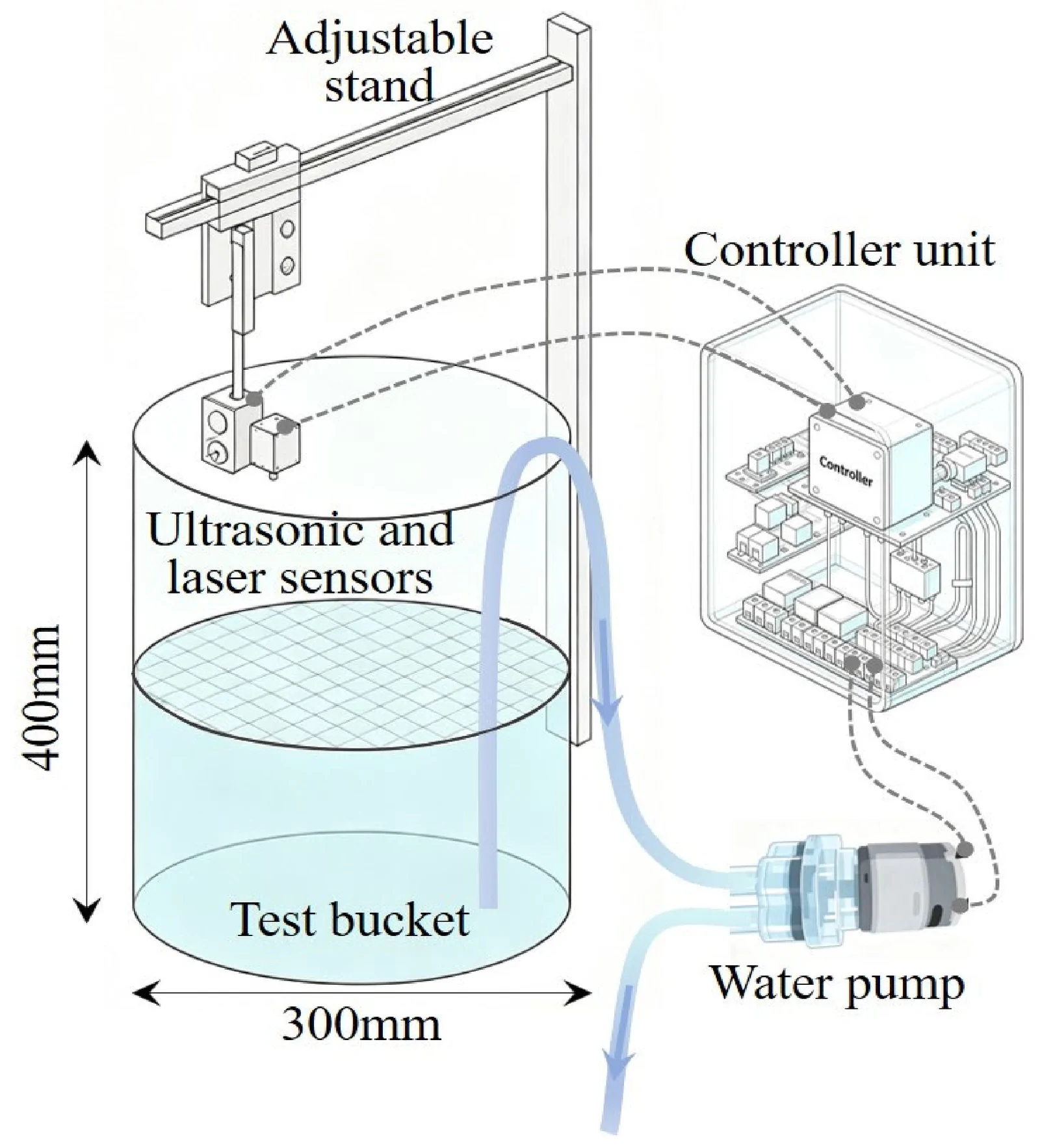
Experimental Platform.

**Figure 3:**
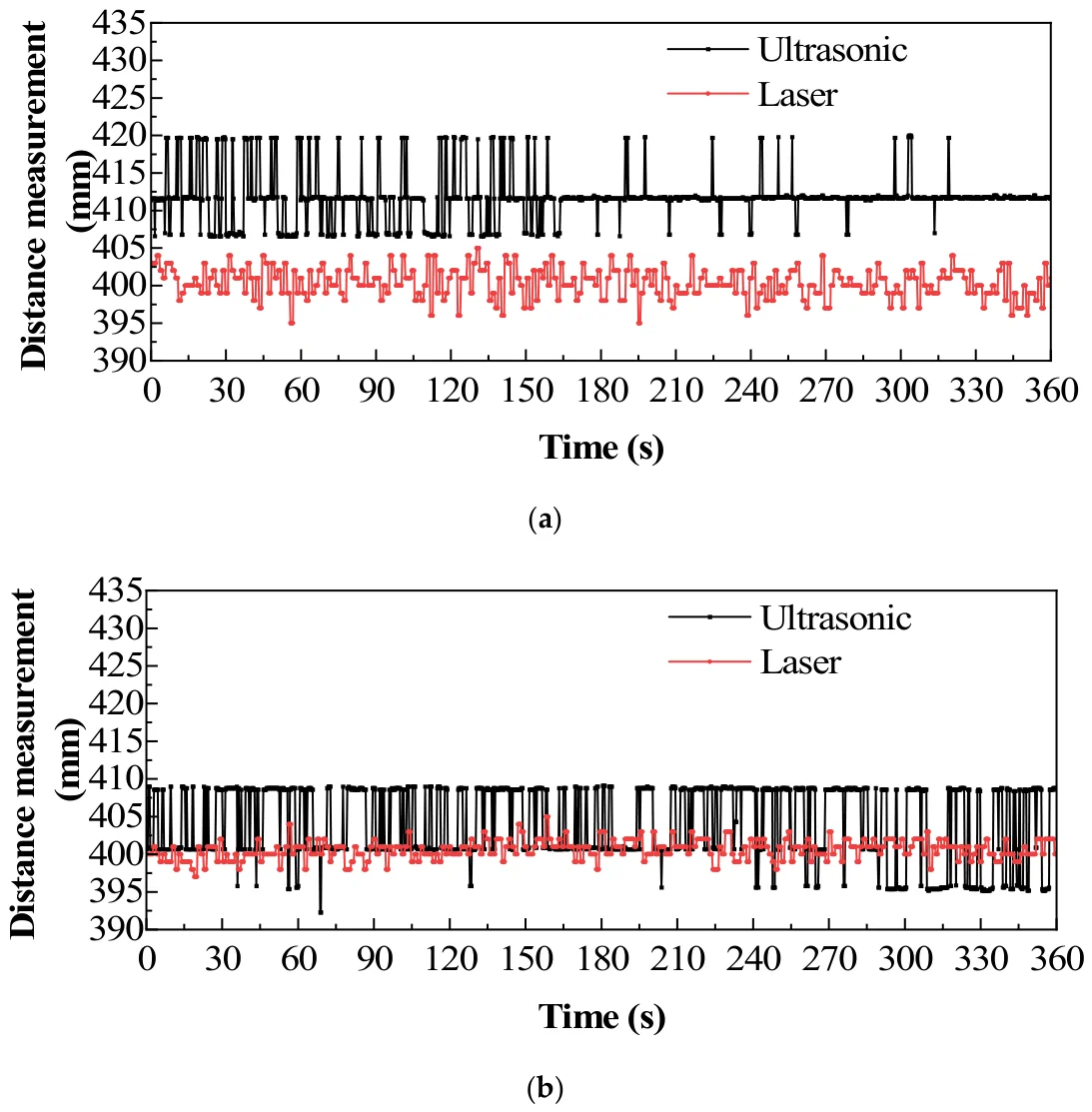

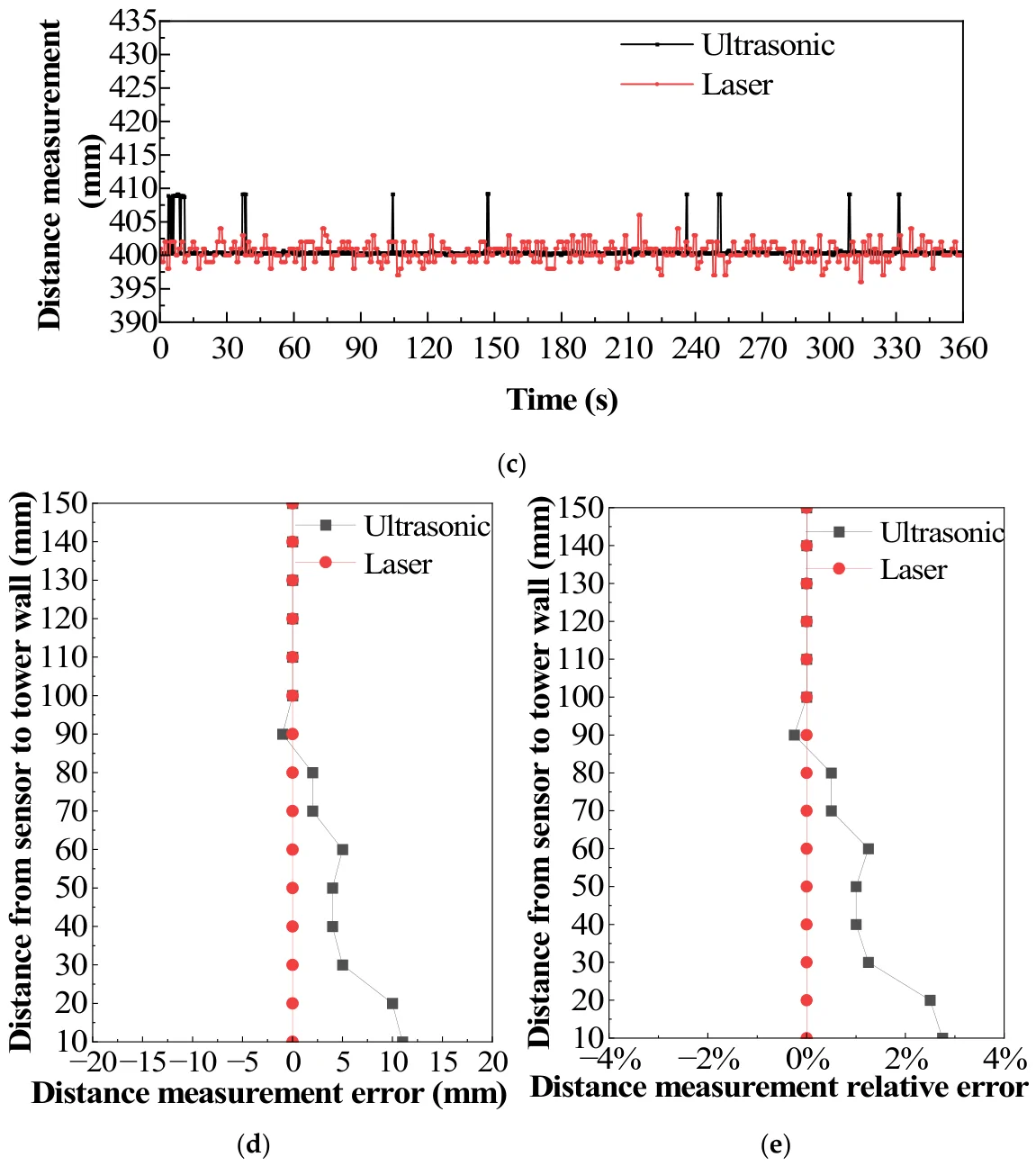
Effect of wall proximity on sensor measurement accuracy: (**a**) Wall distance = 10 mm, (**b**) Wall distance = 50 mm, (**c**) Wall distance = 100 mm, (**d**) *E_D_*; (**e**) *RE_D_*.

**Figure 4:**
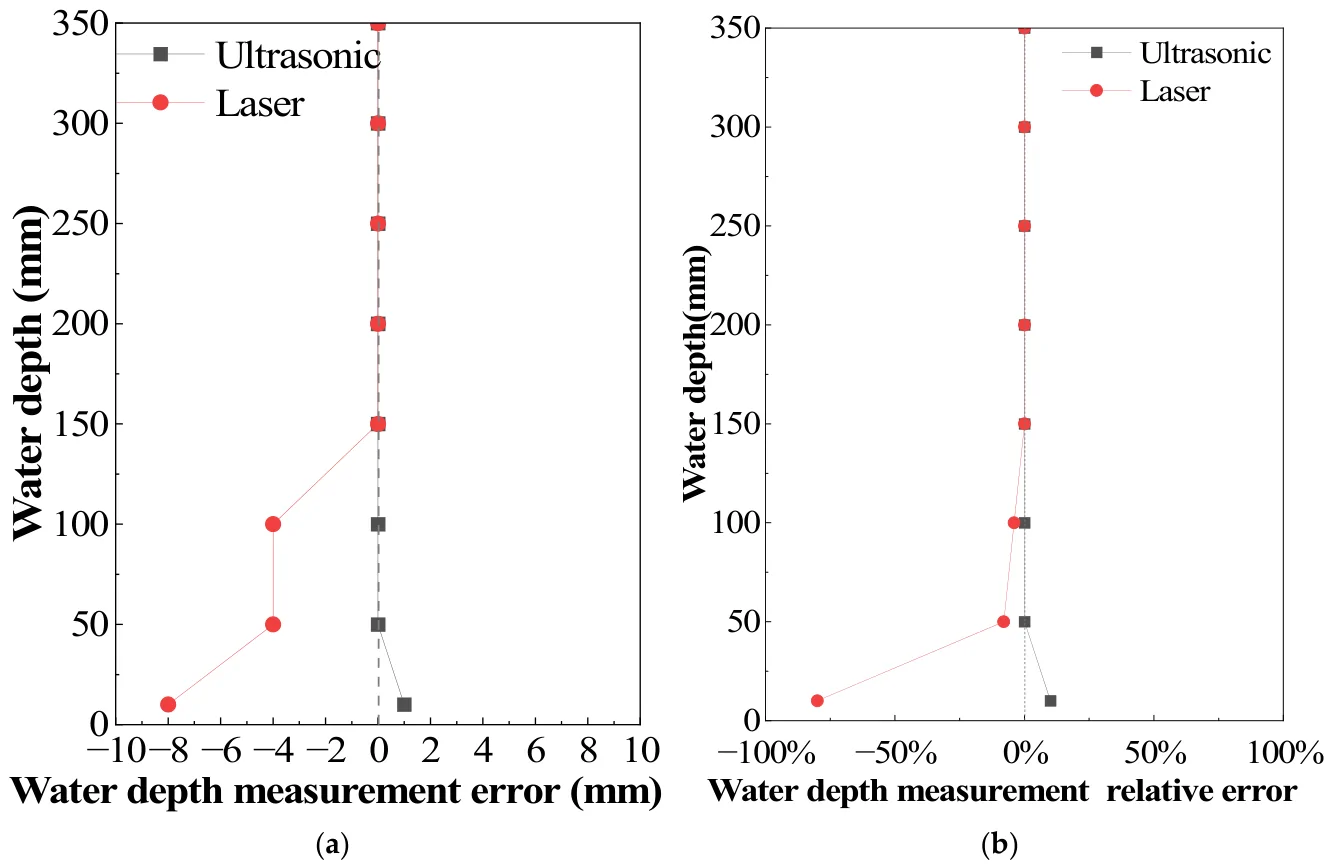
Effect of water depth on sensor measurement accuracy: (**a**) *E_H_*; (**b**) *RE_H_*.

**Figure 5:**
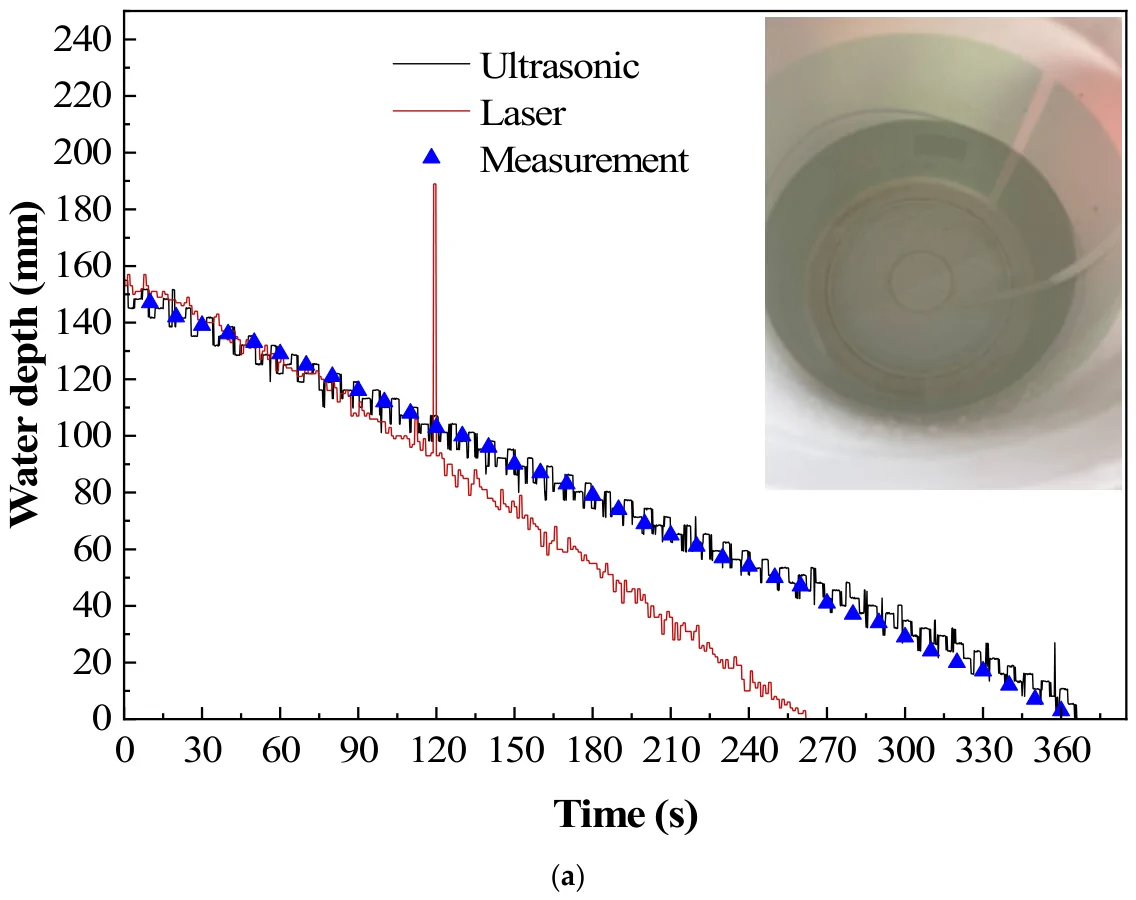

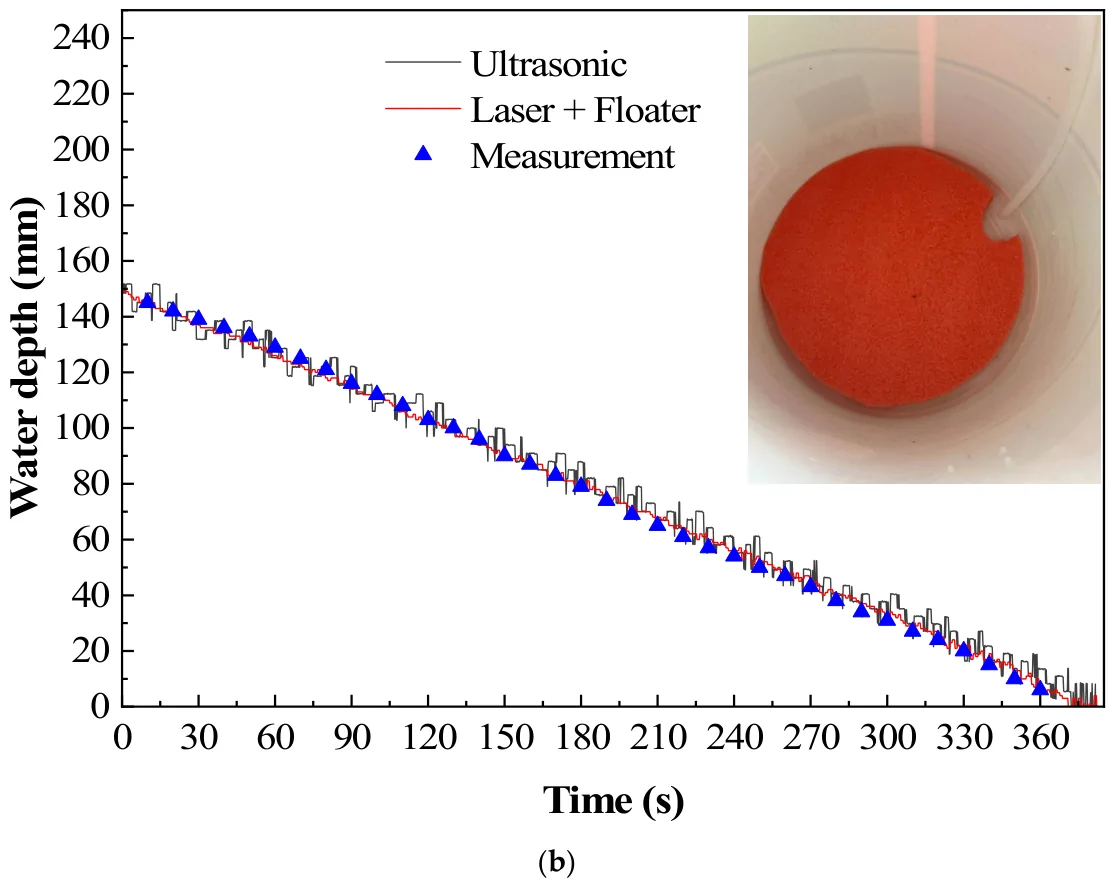
Drainage performance of the ultrasonic sensor, the standalone laser sensor, and the laser sensor with a floating reflector: (**a**) ultrasonic vs. standalone laser; (**b**) ultrasonic vs. laser with floating reflector.

**Table 1:** Statistical analysis of ranging error for wall proximity tests (*n* = 3).

Sensor	Wall Distance (mm)	10	20	30	40	50	60	70	80	90	100	110	120	130	140	150
Ultrasonic	Mean *E*_D_ (mm)	11	10	5	4	4	5	2	2	−1	0	0	0	0	0	0
	SD (mm)	4	4	2	2	2	2	1	1	1	1	1	0	0	0	0
	RMSE (mm)	11	11	5	4	5	5	2	2	1	1	1	0	0	0	0
	MAE (mm)	11	10	5	4	4	5	2	2	1	0	0	0	0	0	0
Laser	Mean *E*_D_ (mm)	0	0	0	0	0	0	0	0	0	0	0	0	0	0	0
	SD (mm)	0	0	0	0	0	0	0	0	0	0	0	0	0	0	0
	RMSE (mm)	0	0	0	0	0	0	0	0	0	0	0	0	0	0	0
	MAE (mm)	0	0	0	0	0	0	0	0	0	0	0	0	0	0	0

**Table 2:** Statistical analysis of water depth error for depth tests (*n* = 3).

Sensor	Water Depth (mm)	10	50	100	150	200	250	300	350
Ultrasonic	Mean *E*_H_ (mm)	1	0	0	0	0	0	0	0
	SD (mm)	1	1	0	0	0	0	0	0
	RMSE (mm)	1	1	0	0	0	0	0	0
	MAE (mm)	1	1	0	0	0	0	0	0
Laser	Mean *E*_H_ (mm)	−8	−4	−4	0	0	0	0	0
	SD (mm)	2	2	2	0	0	0	0	0
	RMSE (mm)	8	4	4	0	0	0	0	0
	MAE (mm)	8	4	4	0	0	0	0	0

## Data Availability

The original contributions presented in this study are included in the article. Further inquiries can be directed to the corresponding author.
